# Surface modification of 3D‐printed polycaprolactone scaffolds with silk fibroin for bone tissue engineering

**DOI:** 10.1002/2211-5463.70366

**Published:** 2026-11-09

**Authors:** Hamed Koeini, Esmaeil Biazar, Davood Zaeifi, Reza Zeinali, Saeed Heidari‐Keshel, Amimasoud Hassanour, Helia Saheli, Arezou Mehrabi

**Affiliations:** ^1^ Biomaterials and Tissue Engineering Group, Department of Biomedical Engineering, To.C. Islamic Azad University Tonekabon Iran; ^2^ Department of Medical Biotechnology and Nanotechnology, Faculty of Medicine Mashhad University of Medical Sciences Mashhad Iran; ^3^ Group of Molecular and Industrial Biotechnology, Department of Chemical Engineering Universität Politècnica de Catalunya Terrassa Spain; ^4^ Hematopoietic Stem Cell Research Center Shahid Beheshti University of Medical Sciences Tehran Iran; ^5^ Department of Medicine, To.C. Islamic Azad University Tonekabon Iran

**Keywords:** 3D printed scaffold, bone tissue engineering, polycaprolactone, silk fibroin, surface modification

## Abstract

One innovative design for tissue‐engineering scaffolds uses 3D printing technology. Here, we used polycaprolactone (PCL) filament as a biocompatible material to design a porous 3D scaffold for bone tissue engineering. After surface modification with silk fibroin, the samples were analyzed using various techniques, including structural characterization, morphological analysis, wettability assessment, cytotoxicity testing, and cell adhesion assays. Scanning electron microscope images showed that the samples were successfully fabricated and exhibited a well‐structured porous architecture. Structural analysis confirmed the presence of a silk fibroin coating on the surfaces of the PCL filaments. Contact angle measurements showed that the control PCL sample, with a contact angle of 73°, decreased to approximately 52° after surface modification with silk fibroin, indicating enhanced hydrophilicity of the scaffolds postmodification. The MTT assay results demonstrated that the modified samples exhibited no cytotoxicity, while electron microscopy images showed good cell adhesion. In general, the surface modification of 3D‐printed PCL scaffolds with silk fibroin represents a promising approach for developing suitable scaffolds for bone tissue engineering applications.

## Graphical abstract

Coating 3D‐printed PCL scaffolds with silk fibroin can improve biocompatibility and bone cell adhesion.

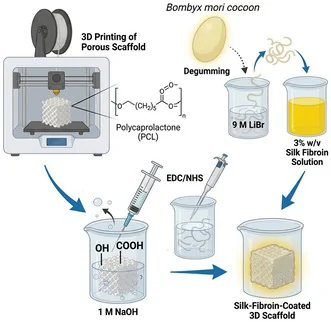

**Abbreviations**
3D: three‐dimensional
ALP: alkaline phosphatase
CAD: computer‐aided design
CO_2_
: carbon dioxide
DMEM‐F12: Dulbecco's Modified Eagle Medium/Nutrient Mixture F‐12
EDC: 1‐ethyl‐3‐[3‐dimethylaminopropyl] carbodiimide
FBS: fetal bovine serum
FTIR: Fourier transform infrared (spectroscopy)
kDa: kilodalton
LiBr: lithium bromide
MG63: human osteosarcoma‐derived osteoblast‐like cell line
MTT: 3‐(4,5‐dimethylthiazol‐2‐yl)‐2,5‐diphenyltetrazolium bromide (cell viability assay)
Na_2_CO_3_
: sodium carbonate
NaOH: sodium hydroxide
NHS: N‐hydroxysuccinimide
PCL: polycaprolactone
RUNX2: Runt‐related transcription factor 2
SD: standard deviation
SEM: scanning electron microscopy
SF: silk fibroin

In recent years, 3D printing technology has gained significant traction across various sectors, including public interest, academic research, and industrial applications, marking a transformative shift in how products are designed and manufactured. This evolution has transitioned 3D printing from a mere prototyping and modeling tool into a robust manufacturing technique, remarkably poised to revolutionize the medical industry [1, 2]. The increasing enthusiasm surrounding 3D printing is not merely a trend; it represents a fundamental change in manufacturing paradigms, especially in the biomedical field, where the technology is being harnessed for applications ranging from organ modeling to the production of customized medical devices [3, 4].

One of the most compelling advantages of 3D printing in medical applications is its ability to produce complex and customized structures directly from digital designs. This capability is particularly beneficial for creating organ models and tissue‐engineering scaffolds with intricate, irregular architectures that are often challenging to fabricate using traditional manufacturing methods [1, 5]. For example, producing bio‐models from imaging data enables accurate representations of organs, facilitating surgical planning and education [6]. Moreover, the rapid transition from design to production enabled by 3D printing paves the way for personalized medicine, in which treatments can be tailored to individual patients' anatomical and physiological characteristics [4]. The current applications of 3D printing in the biomedical field can be broadly categorized into three primary areas: bio‐modeling, biomedical devices, and laboratory biomedical platforms. The first area, bio‐modeling, involves creating large biological structures, such as organs, from imaging data. This process not only aids in surgical planning but also enhances the understanding of complex anatomical relationships [1, 6]. For example, liver models have been utilized effectively for preoperative planning, allowing surgeons to visualize and strategize before actual procedures [7]. The accuracy of these models is crucial, as they can significantly impact surgical outcomes and patient safety. The second area encompasses biomedical devices for *in vivo* applications, including 3D‐printed tissue engineering scaffolds and prosthetics. These devices are engineered to temporarily or permanently replace damaged tissues or organs within a living organism [1, 8]. Developing such scaffolds is particularly important in regenerative medicine, where the goal is to restore the function of damaged tissues by integrating biological materials and cells [9]. Recent advancements in materials science have led to the development of bio‐inks that support cell attachment and growth, thereby enhancing the functionality of 3D‐printed constructs [1, 10]. The ability to customize these scaffolds to meet patients' specific needs further underscores the potential of 3D printing in personalized medicine. The third area features laboratory biomedical platforms, such as microfluidic systems, used for molecular diagnostics and functional cell assays [11]. These platforms leverage the precision of 3D printing to create complex fluidic networks that can manipulate small volumes of fluids, enabling high‐throughput screening and analysis [12]. The integration of living cells into these systems allows for more accurate modeling of biological processes, which is essential for drug development and disease modeling [13]. Despite the considerable advancements in biomedical 3D printing aimed at achieving desired 3D architectures, the role of 3D‐printed components is often confined to providing structural support, with their potential as functional biological substrates frequently overlooked [4].

The study aimed to evaluate the effectiveness of silk fibroin surface modification on 3D‐printed polycaprolactone (PCL) scaffolds, with a focus on enhancing their structural, morphological, and biological properties for tissue engineering applications. This objective includes designing the scaffolds, assessing their structural integrity and hydrophilicity, evaluating cytotoxicity, and analyzing cell adhesion. Ultimately, the study aimed to determine the suitability of these modified scaffolds for future tissue‐engineering applications.

## Materials and methods

This study used a method adapted from Biazar et al. [14] for the extraction of silk fibroin from *Bombyx mori* cocoons. Initially, silk was degummed in a sodium carbonate (Na_2_CO_3_) solution, followed by rinsing to remove sericin proteins. The degummed fibers were then added to a 9.3 M lithium bromide (LiBr) solution and incubated at 60 °C for 4 h. The resulting fibroin solution underwent dialysis against deionized water for 72 h to remove low‐molecular‐weight impurities using a 12 kDa cut‐off dialysis membrane. Finally, the solution was centrifuged at 4000 rpm for 10 min, and the supernatant was refrigerated at 4 °C for the subsequent process [14].

### Design of porous scaffolds

A MakerBot Replicator 2 3D printer was used to fabricate three‐dimensional porous scaffolds via an extrusion‐based technique. The printer operates by melting PCL filament, enabling the construction of scaffolds without adhesives and precise control over strut arrangements and pore dimensions. The integrated feeding system uses an E3Dlite extrusion nozzle (0.4 mm diameter) with x‐ and y‐axis movement controlled by stepper motors, while the build platform moves in the z‐direction. The design of the cubic scaffolds was executed using computer‐aided design (CAD) software, specifically SolidWorks and MakerWare. The scaffolds were configured with a layered architecture, measuring 1 cm in diameter and 6.0 mm in height, featuring a strut spacing (d1) of 400 μm. The architecture incorporated an alternating 0° and 90° pattern to create square pores. Fabrication was achieved through a layer‐by‐layer melt extrusion process, with PCL filament extruded at 40 mm·s^−1^ from a nozzle maintained at 115 °C (Fig. 1).

**Fig. 1:**
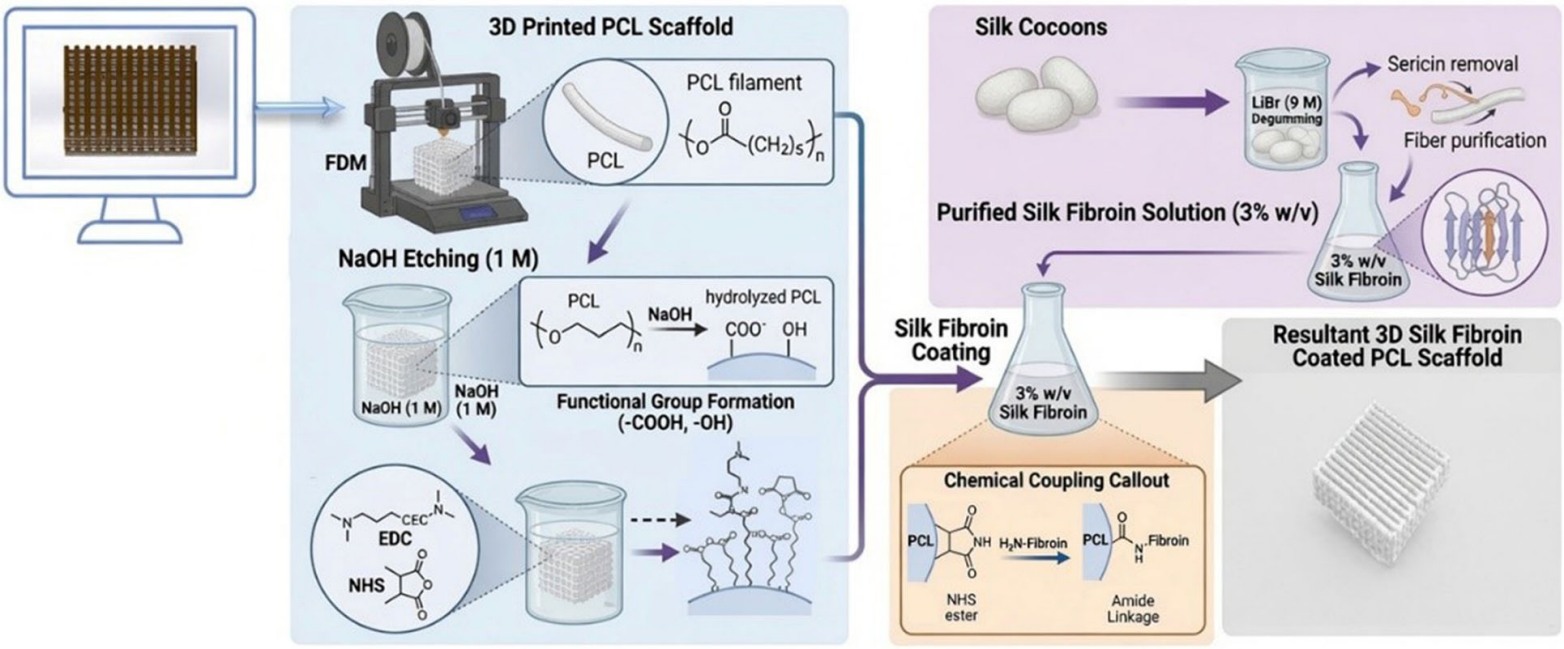
Schematic illustration of the process of designing a 3D printed scaffold and modifying its surface with silk fibroin.

### Scaffold surface modification

Initially, the samples were immersed in a 1 m sodium hydroxide solution for 24 h [15], followed by incubation in a 2 mL solution containing 5 × 10^−3^ g·mL^−1^ of each EDC (1‐ethyl‐3‐[3‐dimethylaminopropyl] carbodiimide) and NHS (N‐hydroxysuccinimide) for another 24 h to facilitate cross‐linking. Then, the treated scaffolds were immersed in 3% silk fibroin solution, followed by reaction for 6 h at room temperature. Subsequently, the primed scaffolds were thoroughly washed with distilled water.

### Morphological surface study

A SEM (PHILIPS XL 30) was employed to examine the surface morphology of the printed scaffold, which was gold‐coated before imaging at a voltage of 20 kV.

### FTIR analysis

Five samples, comprising one control and four modified samples, were analyzed by FTIR using a JASCO 4700 at room temperature, scanning from 450 to 4500 cm^−1^.

### Contact angle analysis

Water droplets were placed on a scaffold (five drops at different points on the surface), and their contact angles were recorded using a contact angle measurement device with digital cameras to determine how well a liquid interacts with a solid surface.

### Cellular studies

#### Culture conditions

MG63 cells (RRID: CVCL_0426) sourced from the Pasteur Institute of Iran. As certified by the supplier, the cell line was authenticated by short tandem repeat (STR) profiling and confirmed to be free of mycoplasma contamination. Cells were maintained in DMEM‐F12 supplemented with 10% fetal bovine serum (FBS) and 1% penicillin/streptomycin at 37 °C in a humidified incubator with 5% CO_2_. Medium was refreshed every 2 days and cells were subcultured at ~70–80% confluence using 0.25% trypsin–EDTA.

#### Cytotoxicity test

Cell proliferation was evaluated using the MTT assay with MG63 cells. The cells were cultured in 96‐well plates at a density of 1 × 10^4^ cells per well, with assessments of viability and proliferation conducted at 24, 48, and 72 h after seeding.

#### Cell adhesion test

To assess cell adhesion and morphology, MG63 cells were cultured on 1‐cm disk‐shaped membranes arranged in 24‐well culture plates. Each membrane received 10 000 cells, maintained in DMEM‐F12 medium enriched with 10% FBS and 1% penicillin/streptomycin, at 37 °C in a 5% CO_2_ environment. The culture medium was refreshed approximately every 2 days to ensure optimal growth conditions. Evaluation of samples was performed with a phase‐contrast microscope, followed by 2‐h fixation in a 2.5% glutaraldehyde solution. After 5 min of washing with FBS and dehydration with ethanol, the samples were dried, coated with a thin layer of gold for surface conductivity, and examined with an SEM (Philips, Netherlands: XL30) at 25 kV.

### Statistical analysis

For quantitative analyses, three replicates were performed. Data were presented as mean ± standard deviation (SD). Statistical significance was assessed using *P*‐values <0.05 for within‐group comparisons and <0.01 for between‐group comparisons, with *t*‐tests and two‐way ANOVA followed by multiple‐comparison tests.

## Results

In this study, SEM was used to assess porosity, pore‐size uniformity, and pore connectivity. As shown in Fig. 2, the 3D‐printed polymer scaffold exhibited uniform pore sizes, precise design, and good pore connectivity, all of which are crucial for creating an ideal scaffold for tissue engineering. Figure 2B and C show the modified samples coated with silk fibroin. The coating is clearly visible, and importantly, the structure's morphology remained intact, with the pore size remaining approximately 350 micrometers.

**Fig. 2:**
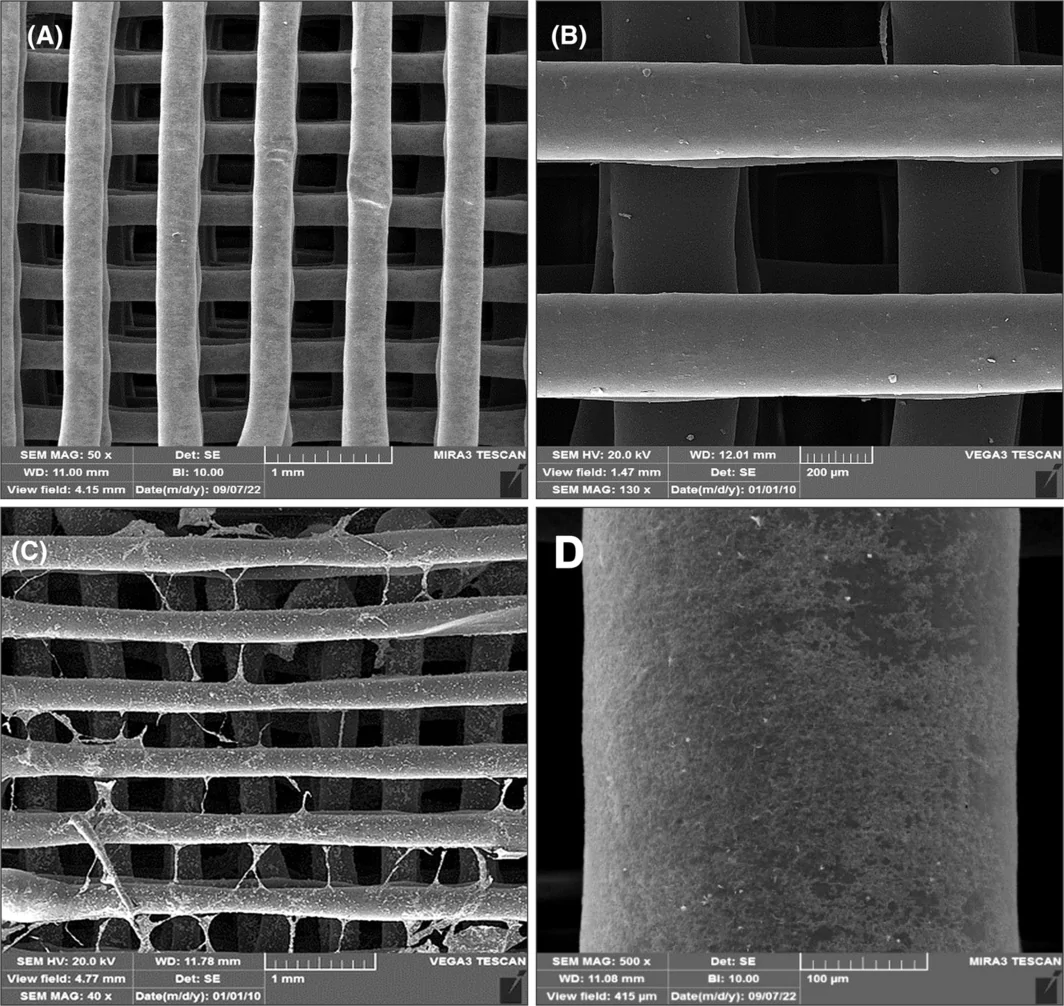
Scanning electron microscopy (SEM) images of the 3D printed scaffold without the modification (A and B), and modified with silk fibroin (C and D).

The FTIR spectrum analysis of unmodified PCL fibers in Fig. 3A reveals characteristic peaks at 2950 and 2820 cm^−1^ for C‐H stretching and a C=O stretching peak at 1725 cm^−1^. Additional peaks at 1188 cm^−1^ (C–O–H stretching) and 1163 cm^−1^ (C–O–C stretching) were also identified.

**Fig. 3:**
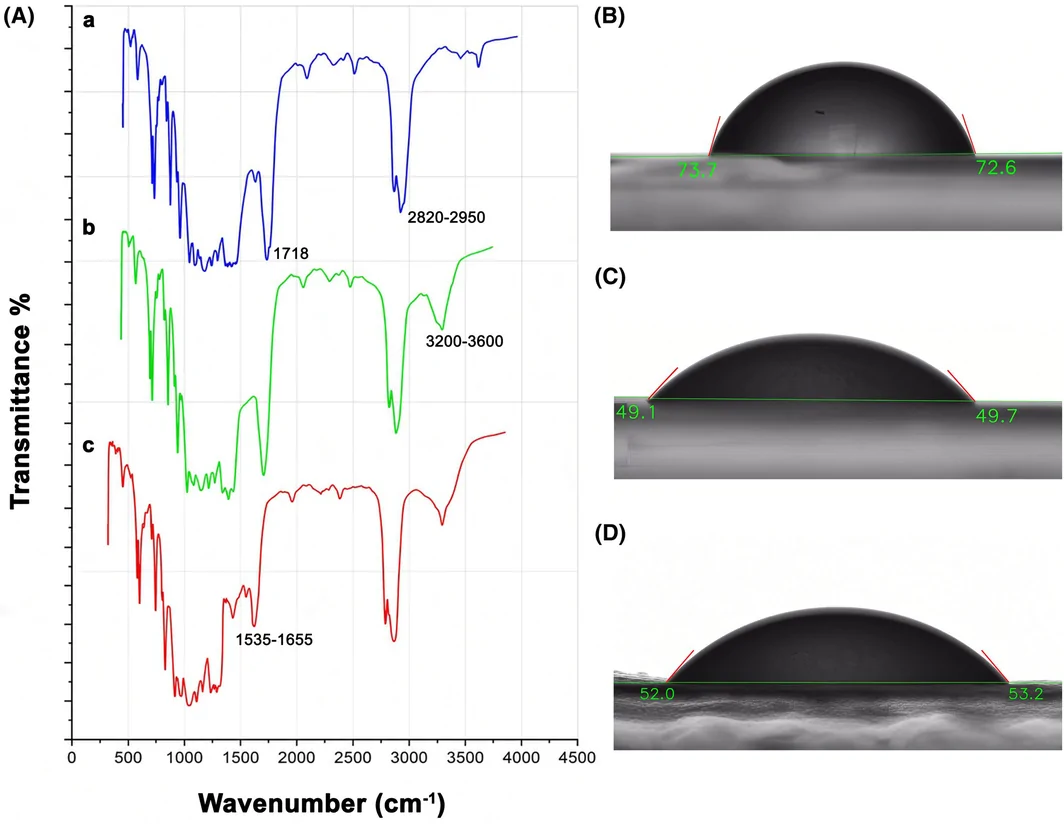
(A) The Fourier transform infrared (FTIR) spectroscopy of neat polycaprolactone (PCL) (A), NaOH‐treated PCL (B), and silk fibroin‐modified PCL scaffolds (C), The contact angles of neat PCL (B), NaOH‐treated PCL (C), and silk fibroin‐modified PCL scaffolds (D).

The modification of PCL fibers with NaOH preserved the characteristic peaks, indicating enhanced hydrophilicity conferred by the ‐OH groups. According to the spectrum of SF (Figure 3A), the vibration band at 1654.92 cm^−1^ demonstrated the presence of amide I (C=O stretching band), and the characteristic vibration bands at 1535.34 and 1238.3 cm^−1^ are attributed to amide II (secondary N–H bending) and amide III (C–N and N–H functionalities), respectively.

The wettability analysis of PCL samples reveals significant changes in hydrophilicity following chemical modifications. The control sample, composed solely of PCL, exhibited a contact angle of 72–74° (Fig. 3B), indicating low hydrophilicity. However, upon treatment with NaOH, the PCL filament structure altered, enhancing its surface properties. The contact angle of the NaOH‐modified sample decreased to 49° (Fig. 3C), indicating improved wettability due to the formation of ‐OH groups on the filament surface. Further modifications involved exposing the NaOH‐treated samples to EDC, NHS, and fibroin. This resulted in a contact angle of 52 degrees for the fibroin‐modified scaffold, which, while slightly higher than that of the NaOH‐modified sample, still indicated good hydrophilicity (Fig. 3D). The increase in contact angle suggests successful cross‐linking of silk fibroin‐coated samples and demonstrates its suitability for further applications.

Figure 4A presents MTT assay results, with pure polycaprolactone as the control. The NaOH‐modified PCL filament coated with silk fibroin showed acceptable biocompatibility. Cultured on TCPS (Tissue Culture Polystyrene) plates at three time points, all surface‐modified samples demonstrated good biocompatibility, indicating their potential for biomedical applications.

**Fig. 4:**
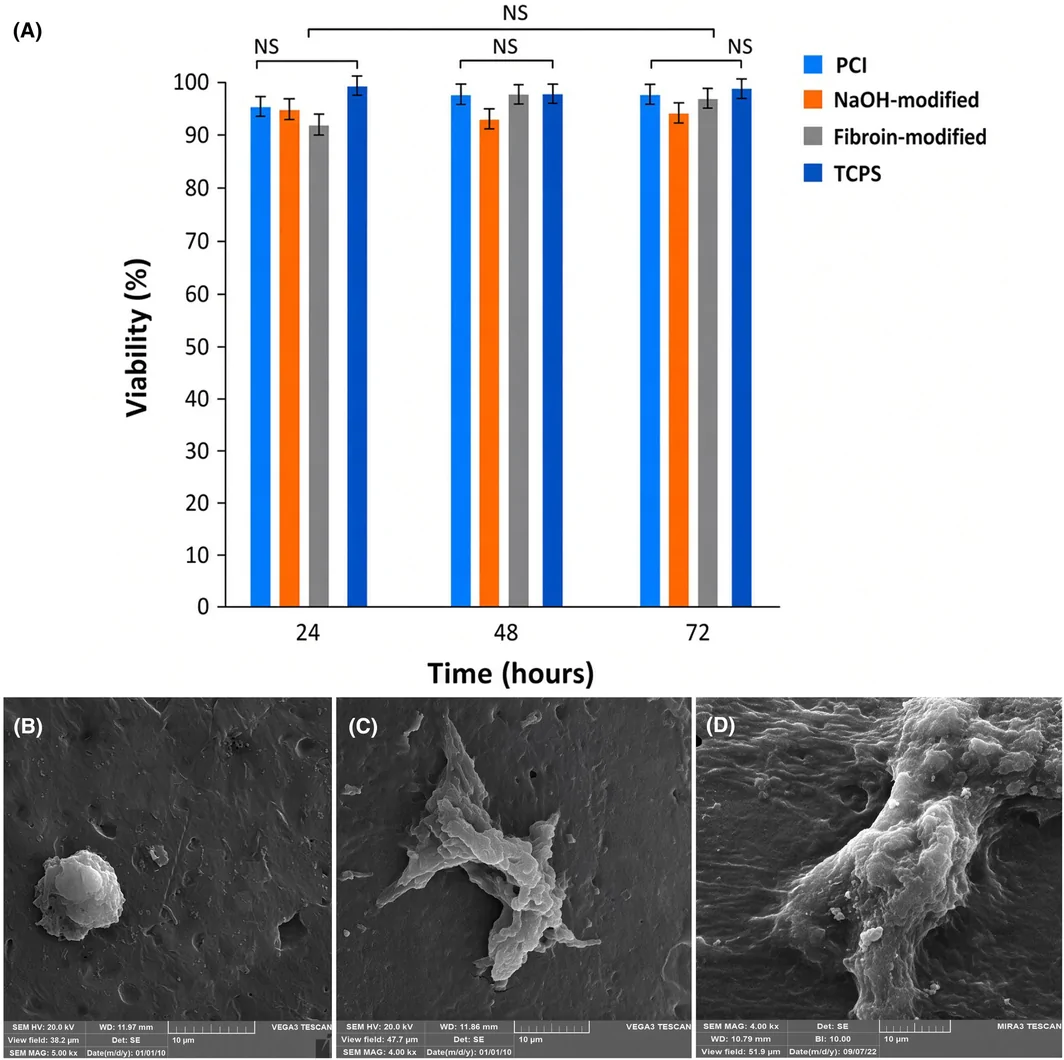
(A) MG63 cell viability by MTT assay at 24, 48, and 72 h on neat PCL, NaOH‐modified PCL, fibroin‐modified PCL, and TCPS control. Data are presented as mean ± SD, *n* = 3 independent replicates. Statistical analysis was performed by two‐way ANOVA followed by Tukey's multiple‐comparison test; NS, not significant (*P* > 0.05). Neat polycaprolactone (PCL) (B), NaOH‐treated PCL (C), and silk fibroin‐modified PCL scaffolds (D).

Cell adhesion on various scaffold surfaces was assessed using electron microscopy postculture. Neat polycaprolactone samples showed low cell adhesion, as depicted in Fig. 4B. In contrast, silk fibroin‐coated samples demonstrated significantly higher cell density and adhesion, illustrated in Fig. 4D. These findings highlight the substantial influence of surface modification on improving cell adhesion and viability on scaffolds.

## Discussion

The surface modification strategy employed in this study, involving NaOH‐induced hydrolysis of PCL followed by EDC/NHS‐mediated covalent immobilization of silk fibroin, represents a rational and effective approach to overcome the inherent hydrophobicity and limited bioactivity of 3D‐printed polycaprolactone scaffolds for bone tissue engineering applications. This method directly addresses a major limitation of melt‐extruded PCL constructs, whose smooth surfaces and high contact angles hinder protein adsorption and initial cell attachment [1, 5].

A significant finding of this work is the preservation of the highly interconnected porous architecture after surface modification. Maintaining pore sizes around 350 μm is particularly advantageous for bone tissue engineering, as this range optimally supports cell attachment and nutrient diffusion/angiogenesis while maintaining adequate mechanical integrity of the scaffold [16, 17]. The fact that the aqueous‐based coating process did not cause pore occlusion or structural collapse highlights the gentle and controlled nature of the modification protocol and its compatibility with the precision of extrusion‐based 3D printing. The reduction in water contact angle from approximately 73° in unmodified PCL to 52° after silk fibroin immobilization indicates a clear improvement in surface hydrophilicity. Moderately hydrophilic surfaces in this range are known to enhance the adsorption of cell‐adhesive proteins such as fibronectin and vitronectin from the surrounding medium, thereby facilitating integrin‐mediated cell attachment and spreading [18, 19]. The observed increase in hydrophilicity compared with neat PCL, while slightly higher than the NaOH‐treated samples alone, is consistent with the successful covalent attachment of a conformal silk fibroin layer via amide bond formation. The MTT assay results demonstrated that silk fibroin‐modified scaffolds exhibited no cytotoxicity toward MG63 cells over 72 h, indicating excellent biocompatibility of the immobilized protein layer. Silk fibroin, prepared using the established degumming and dissolution protocol, is well recognized for supporting cell viability and metabolic activity due to its favorable biochemical properties [14]. This finding is further supported by recent work from our group on carbodiimide‐crosslinked silk fibroin‐based constructs, which similarly demonstrated high cytocompatibility and promoted cell growth [20].

Scanning electron microscopy revealed significantly improved cell adhesion and spreading on the silk fibroin‐modified surfaces compared with unmodified PCL. This enhancement can be attributed to the combined effects of increased surface wettability and the presence of bioactive domains within silk fibroin that promote cell–matrix interactions. Similar improvements in cell attachment have been consistently reported when silk fibroin is used as a surface coating or scaffold component in tissue engineering applications [14, 20].

Importantly, the use of the bone cell model in the present work provides a broad initial biocompatibility screen, as MG63 cells are widely recommended for *in vitro* cytotoxicity testing of biomaterials for bone tissue engineering [21, 22]. However, dedicated follow‐on studies employing osteoprogenitor populations will be required to confirm upregulation of osteogenic markers, including alkaline phosphatase (ALP) activity, RUNX2 expression, and calcium deposition, and to evaluate the coating's influence on osteogenic lineage commitment [23, 24, 25].

While the current *in vitro* results are promising, several aspects require further investigation before clinical translation. Long‐term degradation behavior, mechanical properties under load‐bearing conditions, and performance in dynamic culture systems were not assessed in this study. Moreover, although the covalent attachment strategy ensures coating stability, the influence of the silk fibroin layer on scaffold degradation kinetics and ion release profiles over extended periods remains to be elucidated. Ultimately, *in vivo* evaluation in critical‐sized bone defect models will be essential to determine vascularization, new bone formation, and osseointegration.

## Conclusion

Surface modification offers a materials engineering strategy to tune the surface properties of 3D‐printed PCL scaffolds without compromising their underlying porous architecture. The reduction of the water contact angle from 73° to approximately 52° indicates enhanced surface wettability, which may facilitate interactions between the scaffold surface and the biological environment. These findings, combined with the absence of detectable cytotoxicity and the observed cell attachment, suggest that functionalization with silk fibroin can improve the biological interface of PCL scaffolds while preserving the structural advantages of additive manufacturing. According to these findings, the 3D polycaprolactone scaffolds modified with silk fibroin are potential candidates for tissue engineering.

## Conflicts of interest

The authors declare no conflicts of interest.

## Author contributions

EB conceived and designed the project and supervised and administered the project, SHK performed investigation and methodology, HK performed formal analysis, DZ and AM performed data curation and validation, DZ and RZ wrote the original draft, EB, DZ, AH and HS reviewed and edited the paper, EB and DZ performed visualization.

## Data Availability

The data used to support the findings of this study are available from the corresponding author upon request.
